# Gateway Selection in Millimeter Wave UAV Wireless Networks Using Multi-Player Multi-Armed Bandit

**DOI:** 10.3390/s20143947

**Published:** 2020-07-16

**Authors:** Ehab Mahmoud Mohamed, Sherief Hashima, Abdallah Aldosary, Kohei Hatano, Mahmoud Ahmed Abdelghany

**Affiliations:** 1 Electrical Engineering Department, College of Engineering, Prince Sattam Bin Abdulaziz University, Wadi Addwasir 11991, Saudi Arabia; abdelghany@mu.edu.eg; 2 Electrical Engineering Department, Faculty of Engineering, Aswan University, Aswan 81542, Egypt; 3 Computational Learning Theory Team, RIKEN-Advanced Intelligent Project, Fukuoka 819-0395, Japan; sherief.hashima@riken.jp (S.H.); hatano@inf.kyushu-u.ac.jp (K.H.); 4 Engineering and Scientific Equipment’s Department, Egyptian Atomic Energy Authority, Cairo, Inshas 13759, Egypt; 5 Department of Computer Science, Prince Sattam bin Abdulaziz University, As Sulayyil 11991, Saudi Arabia; ab.aldosary@psau.edu.sa; 6 Faculty of Arts and Science, Kyushu University, Fukuoka 819-0395, Japan; 7 Electrical Engineering Department, Faculty of Engineering, Minia University, Minia 61519, Egypt

**Keywords:** unmanned aerial vehicles, millimeter wave, machine learning, multi-armed bandit

## Abstract

Recently, unmanned aerial vehicle (UAV)-based communications gained a lot of attention due to their numerous applications, especially in rescue services in post-disaster areas where the terrestrial network is wholly malfunctioned. Multiple access/gateway UAVs are distributed to fully cover the post-disaster area as flying base stations to provide communication coverage, collect valuable information, disseminate essential instructions, etc. The access UAVs after gathering/broadcasting the necessary information should select and fly towards one of the surrounding gateways for relaying their information. In this paper, the gateway UAV selection problem is addressed. The main aim is to maximize the long-term average data rates of the UAVs relays while minimizing the flights’ battery cost, where millimeter wave links, i.e., using 30~300 GHz band, employing antenna beamforming, are used for backhauling. A tool of machine learning (ML) is exploited to address the problem as a budget-constrained multi-player multi-armed bandit (MAB) problem. In this setup, access UAVs act as the players, and the arms are the gateway UAVs, while the rewards are the average data rates of the constructed relays constrained by the battery cost of the access UAV flights. In this decentralized setting, where information is neither prior available nor exchanged among UAVs, a selfish and concurrent multi-player MAB strategy is suggested. Towards this end, three battery-aware MAB (BA-MAB) algorithms, namely upper confidence bound (UCB), Thompson sampling (TS), and the exponential weight algorithm for exploration and exploitation (EXP3), are proposed to realize gateways selection efficiently. The proposed BA-MAB-based gateway UAV selection algorithms show superior performance over approaches based on near and random selections in terms of total system rate and energy efficiency.

## 1. Introduction

The use of unmanned aerial vehicles (UAVs), commonly known as drones, gained a lot of consideration in recent years from both academia and industry [1,2]. UAVs are heavily used for military and commercial applications. Leveraging UAVs for future applications looks promising solutions. This is due to their unique properties like flying ability, usability, survivability, functionality, and maneuverability [1,2]. Parts of these applications are data collections, delivery services, environmental monitoring, rescue operations, disaster management, aerial photography, traffic and control monitoring, and wireless communications [1,2]. In this paper, we will focus on the wireless communication applications of the UAVs, particularly for a post-disaster area coverage scenario. Mainly, UAVs can be cost-effective flying aerial base stations (BSs) that can provide coverage to the users in remote and post-disaster areas [3]. They can also be used as on-demand airborne relays connecting a remote user and a cellular BS separated by significant obstacles [4]. For wireless sensor networks (WSNs), UAVs can be utilized to disseminate/collect control and data information from ground-deployed wireless sensors [5,6]. For mobile ad-hoc networks (MANETs), such as vehicular ad-hoc networks (VANETs), UAVs can assist the management and control of VANETs and extend their scalability and coverage [7]. Cache-enabled UAVs can significantly enhance network cashing functionality empowered by the ability of UAVs to track users’ mobility and predict their content requests [8]. Wireless backhaul can be backed up using cost-effective flying UAVs when the wired backhaul link is damaged or needs maintenance. For future fifth-generation (5G) and beyond 5G (B5G) wireless networks, UAVs can play a significate role in enabling and boosting their performance [9]. UAVs can be parts of 5G/B5G heterogeneous networks through distributing on-demand UAV BSs to cover hotspot areas or highly populated events [9]. Moreover, UAVs can highly densify the 5G/B5G networks through the deployment of multiple UAV base stations. 

On the other side, the use of millimeter wave (mmWave), i.e., 30~300 GHz, communications gained a lot of attention due to their large swath of available spectrum, enabling multi-gigabit per second (Gbps) connectivity [10,11,12]. However, mmWave is susceptible to harsh propagation losses due to its high operating frequency in addition to the influence of path blockage [10]. This can be overwhelmed by using directional communication through antenna beamforming, thanks to the high number of packed antenna elements [11]. Accordingly, mmWave coverage is limited to be within a few meters around the mmWave transmitter, which mandates the use of relaying to extend its coverage range [13]. Integrating millimeter wave (mmWave) band, 30~300 GHz, communications with UAV BSs can sustain 5G/B5G requirements due to the large available bandwidth [14,15]. Moreover, UAVs can address many of mmWave challenges, such as the construction of autonomous mmWave relays [16]. 

In this paper, a post-disaster area where the terrestrial network is completely malfunctioned or destroyed was considered to help the surviving people inside it. In this catastrophic situation, several UAVs were distributed to cover this post-disaster area for rescue services adequately. MmWave was employed for the communication links among the UAVs to provide ultra-high-speed Gbps backhaul connections to support critical rescue services such as taking high-resolution videos/photos to the catastrophic area. This was to help in conducting principal analysis and precisely finding out the locations of the victims. The low data rates’ frequency bands may not support these crucial functionalities. Due to the short transmission range of the mmWave signal, some of the UAVs were to operate as access UAVs, providing data connections to the victims/rescue workers, collecting essential information about the post-disaster area such as photographs, and disseminating critical instructions to the victims/rescue workers. Whereas, the other UAVs were to act as gateways relaying information to/from the access UAVs from/to the nearest survival cellular networks, respectively.

In this paper, access UAVs were to select and then fly towards the gateway UAVs, maximizing their achievable data rates while considering the battery cost of their flights. Although the access UAVs could directly fly towards the standing cellular networks, the use of gateway UAVs relaxed the budget of access UAV flights; mmWave in particular was characterized by small coverage. This highly contributes to saving access UAVs’ energy for more rescue operations. The challenge of this gateway UAV selection problem, which is firstly introduced in this paper to the best of our knowledge, comes from its adversarial setting. This is because an access UAV has no prior experience with the data rate gained from connecting with a specific gateway UAV unless it flies and connects with it.

Additionally, this available data rate is influenced by the other access UAV selections due to mutual interference and the time-sharing schedule. In these fully decentralized settings, no prior information is either available or exchanged among UAVs. Despite its realism, this problem is unique and utterly different from the existing UAV gateway/relay selection problems [17,18,19,20,21,22], where UAVs can easily exchange information among them through the fully connected UAV network. Data rates among UAVs can also be anticipated beforehand via prior channel measurements and estimations. Yet, the considered UAV gateway selection problem aims to not only maximize the achievable data rates of the access UAVs, but to also minimize the battery cost of their flights towards the selected gateways. 

In this paper, a tool of machine learning (ML), specifically online learning, was used to address this optimization problem efficiently [23,24,25]. The motivation behind using online learning comes from its ability to deal with both complex and dynamic environments effectively [26], without any prior information, where an agent learns to enhance its future actions based only on its past actions/observations. Towards this end, the gateway UAV selection problem is formulated as a budget-constrained multi-player multi-armed bandit (MAB) problem [27,28,29]. MAB is a particular type of online learning, where an agent wants to maximize its long-term rewards (minimize regrets) via utilizing its previous best arm selection or investigating new choices, known as the exploitation–exploration tradeoff [27,28,29]. Since MAB techniques work online without any prior knowledge about the environment other than the player’s observations while playing, they are considered as the most appropriate solutions for this deemed problem. From the MAB perspective, an access UAV will act as the player aiming to maximize its long-term average data rate, i.e., reward, constrained by its limited budget of battery capacity. On the other side, the gateway UAVs will act as the arms of the bandit. Due to the fully decentralized setting, access UAVs will interact selfishly and concurrently with the environment and select their appropriate gateway UAVs then fly towards them for establishing the mmWave communication links. Only based on their previous successive observed rewards, access UAVs try to compromise the exploitation–exploration tradeoff, i.e., either exploiting their best-selected gateway UAVs so-far or exploring new ones. In this paper, three MAB algorithms, namely upper confidence bound (UCB) [29], Thompson sampling (TS) [30], and the exponential weight algorithm for exploration and exploitation (EXP3) [31], are modified to address such gateway UAV selection problem. Despite the adversarial setting of the problem and the selfish behavior of the access UAVs, the modified MAB algorithms learn to play actions that enhance the overall system performance, as demonstrated in [24,30] and further discussed in this paper. To the best of our knowledge, it is the first time that gateway UAV selection in a fully decentralized mmWave UAV network is formulated as a budget-constrained multi-player MAB problem and efficiently addressed using modified BA-MAB algorithms. The main contributions of this paper can be summarized as follows:

- The problem of gateway UAV selection in post-disaster area coverage is formulated as an optimization problem aiming to maximize the achievable data rates of the access-gateway-cellular relays subject to the limited remaining battery capacity of the access UAVs. This is done in a fully decentralized setting, where no information is either pre-available or exchanged among UAVs;
- A budget-constrained multi-player MAB model is formulated and introduced. In this model, the access UAVs act as the agents, the gateway UAVs act as the arms of the bandit, and the rewards are the long-term achievable data rates constrained by the limited budget of the battery capacity of the access UAVs;
- Three BA-MAB algorithms, i.e., BA-UCB, BA-TS, and BA-EXP3, are proposed to be exploited by each access UAV to selfishly interact with the environment and select the proper gateway UAVs in this adversarial setting. All access UAVs will select their associated gateway UAVs concurrently, and the MAB algorithms implemented in the access UAVs will learn from their previous observations to proactively enhance the overall performance;
- Extensive numerical analysis is conducted to measure the performance of the proposed MAB-based algorithms under different scenarios and compare their performances with two benchmark approaches based on near and random gateway UAV selections.

The rest of this paper is organized as follows; Section 2 summarizes the related work. Section 3 discusses the UAV system model, including the use of the mmWave link model and previews the gateway UAV optimization problem. In Section 4, the proposed BA-MAB algorithms will be explained, followed by numerical analysis in Section 5. Finally, Section 6 delivers the concluding remarks. 

## 2. Literature Review

An efficient gateway-selection algorithm and management technique is required for flying multi-UAV systems for connection with the global network. In [17], the authors surveyed multi-UAV-based heterogeneous flying ad-hoc networks’ (FANET) structure and protocol architecture. Then, a mixture of distributed gateway-selection algorithms and cloud-based stability-control mechanisms were discussed, supplemented by a range of open challenges. The authors in [18] defined the stability of UAV networks, constructed a network partition model, and designed a distributed gateway selection algorithm with dynamic network partition while considering the practical features of UAV networks. Moreover, the number of gateways is managed according to the system requirements. In [19], an energy-efficient method for gateway selection of UAVs involved in relaying information to the heterogeneous cloud was proposed. The authors also make use of the queuing theory and Lyapunov optimization to solve the power-delay tradeoff. In [20], a UAV-enabled two-way relaying communication between two robot swarms in the absence of communication infrastructures in remote areas or post-disaster rescues was handled. UAV is employed as the relay to expand the communication range between two disconnected ground robot swarms due to its several advantages. In addition, the UAV’s trajectory and power allocation were jointly optimized to maximize the sum-rate of the up and downlinks, where the joint optimization problem is decoupled into two sub-problems to address the non-convexity. In [21], a new UAV node placement technique for multi-UAV relay communication was solved based on the non-linear constraint optimization problem. The authors in [22] introduced downlink non-orthogonal multiple access (NOMA) to a UAV-enabled mobile relaying system. 

All of the above existing research works of UAVs gateway/relay selections considered that UAVs have full knowledge at the time of selection, and the network is fully connected, which is not the case of this paper. Wherein, no prior information is available for the access UAVs at the time of gateways selection, and the network is fully disconnected. Moreover, the present works did not consider the cost of access UAV flights towards their selected gateway UAVs, which will be addressed efficiently throughout this paper. 

ML is a promising technology for efficient solutions to the severe UAV problems caused by their utilization of wireless communication. A full survey of all related research where ML methods have been applied on UAV-based communications to improve practical aspects like channel modeling, resource management, security, and positioning is provided in [32]. Moreover, a review of deep reinforcement learning (DRL) algorithms that address emergency applications in wireless communications such as mmWave, intelligent caching, and UAV scenarios are summarized in [25]. In [33], a distributed sense-and-send protocol was proposed to manage the UAVs for sensing and transmission. Moreover, the authors applied RL to solve main problems like trajectory control and resource management. A DRL-based channel and power allocation framework was suggested in [34] for the UAV-enabled IoT system. In this scheme, the UAV-BS can intelligently allocate uplink channels and the transmit power of IoT nodes for maximizing the energy performance of all IoT nodes. Another UAV control policy based on DRL called the deep deterministic policy gradient (UC-DDPG) was proposed in [35]. UC-DDPG addressed the combined problem of 3D mobility of multiple UAVs and energy recharging arrangements to ensure efficient energy and fair broad region coverage of each user with keeping on the service. The authors in [36] proposed two efficient path planning algorithms based on extended MAB to make a rotary-wing UAV act as a wireless BS in a post-disaster area with unknown user distribution. Their proposed algorithms outperform the helical path, which scans the whole post-disaster area by increasing radius circles. 

Despite the existing applications of ML in UAV wireless networks, all related works did not consider the problem of gateway UAV selection in a fully decentralized mmWave UAV network using budget-constrained multi-player MAB techniques.

## 3. System Model

In this section, we will discuss the network architecture of the mmWave UAV wireless networks in addition to the utilized mmWave link model.

### 3.1. UAV Network Architecture 

**Figure 1:**
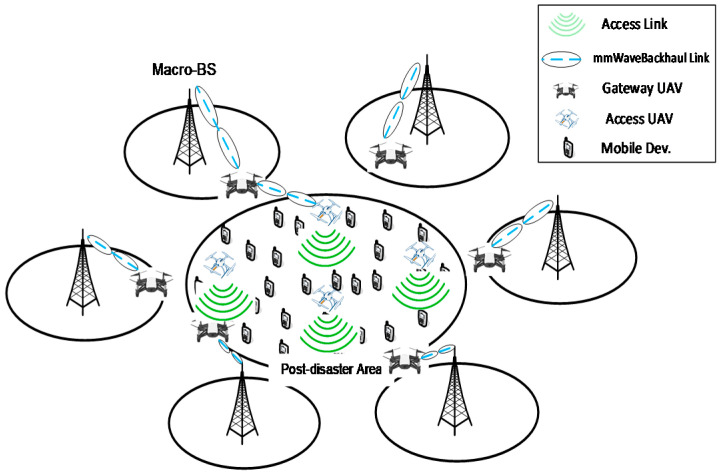
Millimeter wave (mmWave) unmanned aerial vehicle (UAV) network architecture.

Figure 1 shows the considered mmWave UAV network architecture. In this model, there is a post-disaster area, e.g., flood or earthquake areas, in which the cellular macro-BS cellular system is malfunctioned or wholly destroyed. For rescue services, this area will be covered using a group of UAVs. Some of these UAVs will provide the access functionalities inside the catastrophic area, and others will work as gateways for relaying the collected information to the nearest functional cellular macro-BS. To avoid frequent network reconfiguration, the gateway UAVs should have the maximum energy among the other UAVs, while considering their flights to the closest points to the survival cellular macro-BS. Moreover, the network should have alternative gateway UAVs for network presence purposes. The efficient design of UAV network topology via deciding which UAVs should act as access and which should act as gateways considering UAV energy and mobility constraints is beyond the scope of this paper.

The access UAVs provide data connectivity to the victims for essential messaging, and collect valuable information about the post-disaster area using photography. They also collect crucial details about the victims, such as names, ages, genders, photos, locations, etc. Moreover, they disseminate essential instructions to the victims as well as the rescue workers inside the area. High-speed mmWave links are used to connect the access UAVs with the gateway UAVs, and the gateway UAVs with the cellular macro-BSs. The gateway UAVs are directly connected with their associated survival cellular macro-BS without relaying. In this paper, we focus on the backhaul relay links between access UAVs, gateway UAVs, and cellular macro-BSs. After collecting/disseminating the essential information, each access UAV should select and then fly towards one of the gateway UAVs to relay its data to/from the cellular macro-BS through it. It is assumed that the UAV network is not fully connected, i.e., no information can be exchanged among the UAVs unless they fly and connect together. In this paper, we do not consider the fully connected UAV network in order to highly decrease the number of deployed UAVs and relax the need to design an efficient multi-hop routing protocol overcoming the dynamics in the flying UAV network. Moreover, highly complicated route management and maintenance algorithms are needed in the case of a fully connected UAV network to adapt the network configuration when one of the relaying UAVs is out of service, malfunctioning, or needs to be recharged. The design of this fully connected UAV network using a cooperative MAB game, including the required routing protocol in addition to the route management and maintenance algorithms, will be left for our future investigations.

During the flight lifetime of the access UAVs, i.e., during one charging period of their battery, they should collect/deliver as much data as possible. This means that access UAVs should select gateways, maximizing their achievable data rates within the limited battery capacity of their flights. The gateway UAVs are assumed to be only hovering nearby their associated cellular macro-BSs without frequently flying back and forth from them. Thus, the gateway-cellular macro-BS re-association problem due to gateway mobility is relaxed in this paper.

### 3.2. MmWave Link Model

**Figure 2:**
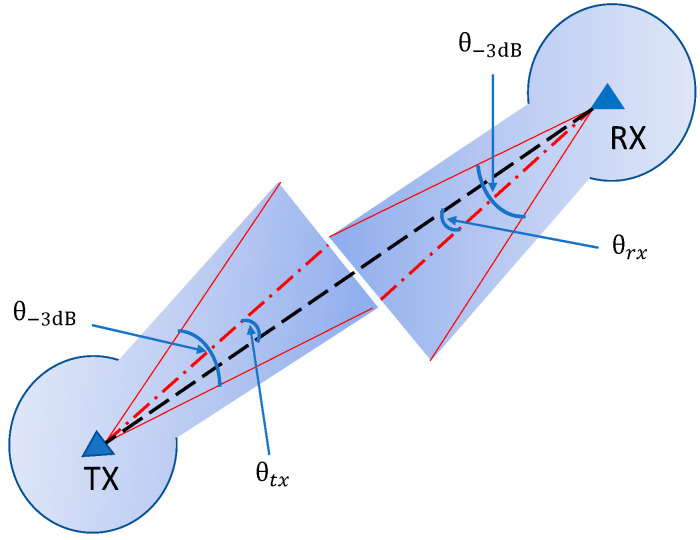
Schematic diagram of the mmWave flat-top antenna model.

In air-to-air communications, the links are almost line-of-sight (LoS). Thus, we will follow the air-to-air mmWave channel model presented in [37] for UAV-to-UAV communication, where the received power at UAV *j* from UAV *i* is expressed as:

(1) $$P_{rx,ij}\left(t\right)=P_{tx,i}G_{tx,ij}\left(\theta_{tx,ij},\theta_{-3\mathrm{dB}}\right)G_{rx,ji}\left(\theta_{rx,ji},\theta_{-3\mathrm{dB}}\right){\left(\frac{\lambda }{4\pi }\right)}^{2}{\left(d_{ij}\right)}^{-\alpha }$$

where $P_{tx,i}$ is the transmit power of UAV *i*, $\lambda =\frac{c}{f}$ is the wavelength, $d_{ij}$ is the separation distance between UAV *i* and UAV *j*, and α is the path loss exponent. $G_{tx,ij}\left(\theta_{tx,ij},\theta_{-3\mathrm{dB}}\right)$ and $G_{rx,ji}\left(\theta_{rx,ji},\theta_{-3\mathrm{dB}}\right)$ refer to the transmitter (TX) and receiver (RX) mmWave beam-forming gains, respectively. $\theta_{tx,ij}$ is the beam offset angle of the TX beam direction to the location of the RX, while $\theta_{rx,ji}$ defines the beam offset angle of the RX beam direction to the location of the TX. $\theta_{-3\mathrm{dB}}$ is the −3dB beam-width. Additionally, in [37], a flat-top antenna model is utilized, in which $G\left(\theta ,\theta_{-3\mathrm{dB}}\right)$ can be expressed as:

(2) $$G\left(\theta ,\theta_{-3\mathrm{dB}}\right)=\{\begin{array}{c} \frac{2\pi -\left(2\pi -\theta_{-3\mathrm{dB}}\right)\varepsilon }{\theta_{-3\mathrm{dB}}},\;\;\;\mathrm{if}\;\left|\theta \right|\leq \frac{\theta_{-3\mathrm{dB}}}{2}\\ \varepsilon \;\;\;\;\;\;\;\;\;\;\;\;\;\;\;\;\;\;\;\;\;\mathrm{Otherwise} \end{array}$$

where ε is the sidelobe gain, 0≤ε≤1. However, any other mmWave beam-forming strategy can be applied in the proposed scheme. Figure 2 shows the schematic diagram of the considered flat-top mmWave antenna model for both mmWave TX and RX. The angles of the TX/RX communication beams, i.e., $\theta_{tx,ij}$ and $\theta_{rx,ji}$, are tuned by means of beam-forming training using steerable antenna arrays in both TX and RX UAVs.

### 3.3. Problem Formulation

In this section, we formulate the optimization problem of the decentralized gateway UAV selection. Suppose that there are *N* access UAVs and *M* gateway UAVs distributed in the post-disaster area, where 1≤i≤N, and 1≤j≤M. Each access UAV *i* should select one of the gateway UAVs, i.e., gateway UAV *j*, then fly towards it for relaying its collected information. This is done at every time t, 1≤t≤T, where T indicates the total lifetime before the battery of the access UAV needs recharging. In this paper, the selected gateway UAVs should maximize the long-term average data rates of the access-gateway-cellular relays while satisfying the battery capacity constraint of the access UAVs during their flight periods. This maximization problem can be formulated as follows:

(3) $$\underset{I\left(1\right),\ldots ,I\left(T\right)}{\max}\frac{1}{T}\underset{t}{\sum }\underset{i,j}{\sum }I_{ij}\left(t\right)\Psi_{ij}\left(t\right)$$

s.t.

(1) $\underset{j}{\sum }I_{ij}\left(t\right)=1,\;1\leq j\leq M,$
(2) $TB_{h}t_{h}+\overset{T}{\underset{t=1}{\sum }}B_{f}\frac{d_{f,ij}\left(t\right)\;}{v_{f}}+\frac{P_{tx}L_{D}}{\Psi_{ij}\left(t\right)}\leq \Xi_{C,}\;1\leq i\leq N$

where $\Psi_{ij}\left(t\right)$ is the data rate of the relay link between access UAV *i*, gateway UAV *j*, and the cellular macro-BS *j* at time t. Let $U=\text{\{}I\in {\left\{0,1\right\}}^{N\times M}\;|\underset{j}{\sum }I_{ij}=1\mathrm{for}\;i=1,\ldots ,N\}.$ For each time t=1,…,T, I(t)∈U is a matrix where $I_{ij}\left(t\right)$ refers to a linkage indicator function that is equal to 1 if the access UAV *i* is linked with gateway UAV *j* and 0, otherwise, where each access UAV *i* should select only one gateway UAV *j* at a time *t* as given in the first constraint of (3). The goal of the optimization problem in (3) is to maximize the long-term average total system rate by optimizing the selection of the linkage matrix I(t). In the second constraint of (3), the simple UAV energy model introduced by the authors in [36] is utilized. However, more sophisticated UAV energy models like that presented in [38] can be adopted in (3) without affecting the generalization of (3). In the second constraint in (3), $B_{h}$ and $\;B_{f}$ are the hovering and flying engine powers in Watts, respectively. $t_{h}$ describes the hovering time needed for an access UAV to gather essential information from its dedicated coverage section. $d_{f,ij}\left(t\right)$ is the minimum distance that should be flown by access UAV *i* to establish the mmWave communication link with the gateway UAV *j* chosen by access UAV *i* at time t, and $v_{f}$ reflects the flying speed of access UAV in m/sec. The term $\frac{P_{tx}L_{D}}{\Psi_{ij}\left(t\right)}$ indicates the energy consumed due to data communication in Joule, where $L_{D}$ indicates the size of transmitted information data in bits, and $\Xi_{C}$ is the access UAV total battery capacity in Joule. Herein, we assume that all access UAVs have the same specifications of $B_{h}$, $B_{f}$, $\Xi_{C}$, $t_{h}$, and $v_{f}$. In (3), we give high priority to the access UAVs’ battery consumptions when trying to select the gateway UAVs, maximizing the achievable data rate. This is because UAV battery consumption is one of the main concerns when designing an efficient UAV network due to its limited capacity, considering the high energy consumed during access UAV flights. However, we did not consider the constraint of the gateway UAV battery capacity due to two main reasons: (1) Typically, gateway UAVs have the highest remaining battery capacity among the UAVs; (2) in the network setting, gateway UAVs will not frequently fly like access UAVs. Instead, they hover beside their associated cellular macro-BS most of the time to provide relaying functionalities. It is stated in [36,38] that the power consumed in UAV hovering is much lower than that consumed during UAV flying. 

Without loss of generality, we assume a half-duplex decode and forward (DF) relay strategy where time resources are equally divided between the access to gateway link and the gateway to cellular macro-BS linkage. Moreover, the uplink scenario is considered with round-robin time-sharing scheduling among access UAVs attached to the same gateway UAV. Thus, $\Psi_{ij}\left(t\right)$ in (3) can be expressed as:

(4) $$\Psi_{ij}\left(t\right)=\frac{1}{2\mu_{j}\left(t\right)}\min\left(r_{ij}\left(t\right),r_{j{\mathrm{BS}}_{j}}\left(t\right)\right),$$

where $\mu_{j}\left(t\right)$ indicates the number of time-scheduled access UAVs connected with the same gateway UAV *j* at time t. $r_{ij}\left(t\right)$ is the achievable data rate of the mmWave link between access UAV *i* and gateway UAV *j*, and $r_{j{\mathrm{BS}}_{j}}\left(t\right)$ reflects the achievable data rate between gateway UAV *j* and its corresponding cellular macro-BS *j*. In this paper, we will focus on the value of $r_{ij}\left(t\right)$ as it mainly results from the interference inside the UAV wireless network coming from other access-gateway selections. $r_{ij}\left(t\right)$ can be expressed as:

(5) $$r_{ij}\left(t\right)=BW\log_{2}\left(1+\gamma_{ij}\left(t\right)\right),$$

where BW is the allocated bandwidth, and $\gamma_{ij}\left(t\right)$ refers to the signal-to-interference plus noise–power ratio (SINR) of the linkage between access UAV *i* and gateway UAV *j* at time t. Based on (1) and considering the uplink scenario, $\gamma_{ij}\left(t\right)$ can be represented as:

(6) $$\gamma_{ij}\left(t\right)=\frac{P_{tx,i}G_{tx,ij}\left(\theta_{tx,ij},\theta_{-3\mathrm{dB}}\right)G_{rx,ji}\left(\theta_{rx,ji},\theta_{-3\mathrm{dB}}\right){\left(\frac{\lambda }{4\pi }\right)}^{2}{\left(d_{ij}\right)}^{-\alpha }}{\overset{\nu \left(t\right)}{\underset{k=1,k\neq i}{\sum }}P_{tx,k}G_{tx,kj}\left(\theta_{tx,kj},\theta_{-3\mathrm{dB}}\right)G_{rx,jk}\left(\theta_{rx,jk},\theta_{-3\mathrm{dB}}\right){\left(\frac{\lambda }{4\pi }\right)}^{2}{\left(d_{kj}\right)}^{-\alpha }+N_{0}},$$

where $N_{0}$ is the noise power, and ν(t) is the number of access UAVs attached to the other gateway UAVs and scheduled within the same time slot assigned by gateway UAV *j* to access UAV *i*.

$r_{j{\mathrm{BS}}_{j}}\left(t\right)$ in (4) can be evaluated using (5), except that the SINR from gateway UAV *j* to its corresponding macro-BS *j* should be applied, i.e., $\gamma_{j{\mathrm{BS}}_{j}}\left(t\right)$, which can be expressed as:

(7) $$\gamma_{j{\mathrm{BS}}_{j}}\left(t\right)=\frac{P_{tx,i}G_{tx,j{\mathrm{BS}}_{j}}\left(\theta_{tx,j{\mathrm{BS}}_{j}},\theta_{-3\mathrm{dB}}\right)G_{rx,{\mathrm{BS}}_{j}j}\left(\theta_{rx,{\mathrm{BS}}_{j}j},\theta_{-3\mathrm{dB}}\right){\left(\frac{\lambda }{4\pi }\right)}^{2}{\left(d_{j{\mathrm{BS}}_{j}}\right)}^{-\alpha }}{\overset{M\left(t\right)}{\underset{h=1,h\neq j}{\sum }}P_{tx,h}G_{tx,h{\mathrm{BS}}_{j}}\left(\theta_{tx,h{\mathrm{BS}}_{j}},\theta_{-3\mathrm{dB}}\right)G_{rx,{\mathrm{BS}}_{j}h}\left(\theta_{rx,{\mathrm{BS}}_{j}h},\theta_{-3\mathrm{dB}}\right){\left(\frac{\lambda }{4\pi }\right)}^{2}{\left(d_{h{\mathrm{BS}}_{j}}\right)}^{-\alpha }+N_{0}},$$

where M(t) is the number of gateway UAVs transmitting simultaneously at time *t*.

Although the problem in (3) can be considered as a binary linear programming (BLP) problem, the conventional solutions of combinatorial optimization, such as the highly complicated exhaustive search approach, the graph-based approach, the branch-and-bound approach, etc., are not feasible solutions to (3). This is because the objective values $\Psi_{ij}\left(t\right)$, corresponding to a candidate linkage matrix I(t), are not known beforehand unless access UAVs fly and connect with their corresponding gateway UAVs in I(t). Thus, in the exhaustive search solution, for example, all $M^{N}$ I(t) access UAV flights and their corresponding linkage matrices $\Psi_{ij}\left(t\right)$ should be obtained before selecting the optimal I(t) configuration. This is infeasible considering the battery capacity constraint of the access UAVs along with the time-sensitive rescue service. This highly complex and dynamic problem motivates us to use online learning by means of the multi-player MAB approach to address it. In this approach, access UAVs time-by-time proactively learn from their previous gateway selections/data rate observations how to enhance their future gateway selections, maximizing their achievable data rates within their limited budget of battery capacity. This is done without any prior knowledge about $\Psi_{ij}\left(t\right)$.

## 4. Proposed Battery-Aware MAB Algorithms 

In this section, we will describe the general concept of MAB as an efficient online learning tool. Then, we will propose three battery-aware budget-constrained multi-player MAB-based algorithms, namely BA-UCB, BA-TS, and BA-EXP3, to address the gateway selection problem mentioned above.

### 4.1. General Single Player MAB Strategy 

The MAB problem is a purely online ML, in which the player strives to gain the maximum reward from multiple arms of slot machines [27,39]. Precisely, the MAB problem aims to detect and select, through finite trials, the arm that maximizes the long-term reward. An assumption is made that the player has no prior information about the reward rates of any of the arms, motivating us to use it as an efficient solution for the gateway selection problem under consideration. In the beginning, the player assembles information on each slot machine (exploration) by trying as many arms as possible then estimating the arm that may have the highest expected reward. Then, the player plays with that arm (exploitation) as much as possible. If the estimation time is long enough, the player can precisely estimate the expected reward of each arm. Meanwhile, if the estimation time is short, the player cannot obtain many rewards, and the player may select the arm with a low reward, leading to unprecise reward results. Based on the rewards distribution, the MAB problem can be classified as *stochastic* or *adversarial*. In the first type, the rewards of the arms are assumed to be drawn from independent and identical distributions (i.i.d.), which are unknown for the player. However, in the second type, the rewards are chosen arbitrarily by the environment. Several MAB-based algorithms have been proposed to deal with the tradeoff of the exploration–exploitation such as UCB, TS, and EXP3. In its typical form, the MAB-based algorithm has *K* possible actions a∈{1,2,…,K} to choose from, i.e., arms and *T* rounds. In each round *t*, the algorithm selects an arm $a_{t}\in \left\{1,2,\ldots ,K\right\}$ and gathers a reward related to the arm, i.e., $r_{a_{t}}$. The algorithm attempts to learn which arm is the best, while not consuming too much exploration time. 

**Figure 3:**
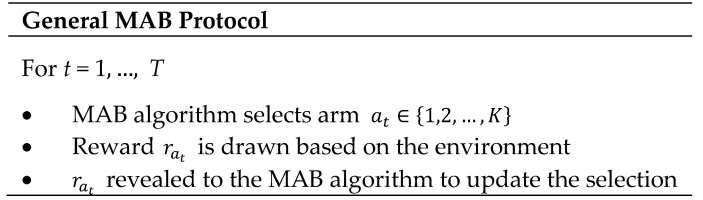
General multi-player multi-armed bandit (MAB) protocol.

Figure 3 summarizes the typical MAB protocol. In this protocol, at every time t, the utilized MAB algorithm, e.g., UCB, TS, EXP3, selects one of the available actions, i.e., arms, then observes the reward resulting from taking that action. This reward will be revealed to the MAB algorithm to enhance its action selection in the next round based on its previous observations up to (but excluding) time t and according to its policy. Typically, regret is used to measure the efficiency of MAB algorithms. It is defined as the loss of the accumulative reward resulting from non-optimal arms selection. Recently, budget-constrained MABs have brought much research attention, where playing an arm requires spending a cost while receiving a reward, and the player aims at maximizing the long-term reward under its limited budget [40]. In this scenario, the player plays for the materialized costs until the remaining budget is exhausted, at which point the algorithm terminates. In the considered problem, the limited budget is the battery capacity of the access UAV, where the access UAV will incorporate in the game until its battery needs for recharging. 

### 4.2. Multi-Player MAB Strategy 

Like the single-player MAB discussed above, in the multi-player MAB, each player chooses an action in successive trials to receive an unknown reward too [26,41,42,43]. If multiple players select the same arm, collisions happen. Based on the collision model, players may share the rewards as in our case where time-sharing scheduling is used, or no one receives the reward as in the case of cognitive radios [41,42,43]. Based on the information exchanged among the players, multi-player MAB can be categorized as centralized or decentralized. In the centralized setting, the game is played collectively among the players via exchanging full observation getting the game looks like a single-player MAB. However, in the case of a decentralized game, no data is transferred among the players, and each player selects his action only based on his own recorded observations. Different from centralized games, collisions are unavoidable in the case of the decentralized category. Thus, each player acts selfishly to learn collision patterns and tries to avoid them while interacting with the environment. In the considered problem, the access UAVs learn to prevent not only collisions but also interference coming from other access–gateway links. Despite this adversarial environment, the authors in [41] showed that the minimum regret of the decentralized multi-player MAB grows at the same rate as the centralized counterpart. The authors in [26] also showed that players in selfish multi-player MAB could learn how to play actions that enhance their rewards and overall system performance as well. Motivated by these reliable results, we adopted a selfish multi-player MAB to address the problem of gateway UAV selection in a fully decentralized setting. Moreover, the battery constraint of access UAV flights was added to the played game.

### 4.3. Proposed Battery-Aware Multi-Player MAB Algorithms

Herein, three BA-MAB algorithms are proposed to be played selfishly and concurrently by the access UAVs to select their corresponding gateway UAVs at every round of selection.

#### 4.3.1. Proposed BA-UCB Algorithm

UCB is one of the famous MAB algorithms that performs tradeoffs of exploitation–exploration very effectively. It tries to increase the confidence of the selected action by decreasing its uncertainty. Algorithm 1 summarizes the proposed BA-UCB algorithm, which is implemented in each access UAV *i* for selfish gateway UAV selection. It is assumed that the access UAV *i* is aware of the flying distances from its current location towards its surrounding gateway UAVs, i.e., $d_{f,ij}\left(t\right)$ in (3). This information can be obtained using GPS sensors attached to the UAVs. At each time t, it is also assumed that each access UAV is aware by its remaining battery capacity $\Xi_{r,i}\left(t\right)$. As an initialization of the algorithm, each UAV *i* will randomly select and fly towards one gateway UAV at once and observe the obtained payoffs $\Psi_{ij}\left(t\right)$ as given in (4). From t=M+1 until t=T, access UAV *i* will select gateway UAV *j*, maximizing the following equation:

(8) $$j^{*}\left(t\right)=\underset{}{\arg\underset{1\leq j\leq M}{\max}\left(E\left(\Psi_{ij}\left(t-1\right)\right)+\sqrt{\frac{2\ln\left(t\right)}{x_{ij}\left(t-1\right)}}-\rho \frac{d_{f,ij}\left(t\right)}{\Xi_{r,i}\left(t\right)\;}\right)}$$

where $E\left(\Psi_{i}^{j}\left(t-1\right)\right)$ indicates the average data rate observed by connecting access UAV *i* with gateway UAV *j* up to (but excluding) time t. $\sqrt{\frac{2\ln\left(t\right)}{x_{ij}\left(t-1\right)}}$ is the exploration term, where $x_{ij}\left(t-1\right)$ reflects the number of times gateway UAV *j* has been selected by access UAV *i* up to (but excluding) time t. The added term $\rho \frac{d_{f,ij}\left(t\right)\;}{\Xi_{r,i}\left(t\right)\;}$ implies the battery cost of access UAV *i* flight to reach gateway UAV *j*, where ρ>0 is a factor for compromising between the achievable data rate and the battery cost. The ratio $\;\frac{d_{f,ij}\left(t\right)\;}{\Xi_{r,i}\left(t\right)\;}$ indicates that more residual battery capacity is needed to reach far gateways with high separation distances $d_{f,ij}\left(t\right)\;$. The policy proposed in (8) compromises between exploiting gateway UAVs having higher observed average data rates associated with minimum battery cost to fly towards it or exploring other low investigated ones. After selecting and flying towards gateway UAV $j^{*}$ at time t and obtaining its corresponding reward $\Psi_{ij^{*}}\left(t\right)$, its number of selections $x_{ij^{*}}\left(t\right)$, average achievable data rate $E\left(\Psi_{ij^{*}}\left(t\right)\right)$, and remaining battery capacity $\Xi_{r,i}\left(t+1\right)$ are updated, as given in steps 2, 3, and 4 in Algorithm 1.
**Algorithm 1:** BA-UCB gateway UAV selection. **Initialization:** Randomly select each gateway UAV *j*, 1≤j≤M at once and their corresponding $\Psi_{ij}\left(t\right)$ are observed, and set the number of selection times $x_{ij}\left(t\right)=1$ for 1≤t≤M **For**
t=M+1:T
Draw a gateway UAV and obtain the reward:$j^{*}\left(t\right)=\underset{}{\arg\underset{1\leq j\leq M}{\max}\left(E\left(\Psi_{ij}\left(t-1\right)\right)+\sqrt{\frac{2\ln\left(t\right)}{x_{ij}\left(t-1\right)}}-\rho \frac{d_{f,ij}\left(t\right)\;}{\Xi_{r,i}\left(t\right)\;}\right)}$Obtain $\Psi_{ij^{*}}\left(t\right)$
$x_{ij^{*}}\left(t\right)=x_{ij^{*}}\left(t-1\right)+1$$E\left(\Psi_{ij^{*}}\left(t\right)\right)=\frac{1}{x_{ij^{*}}\left(t\right)}\overset{x_{ij^{*}}\left(t\right)}{\underset{h=1}{\sum }}\Psi_{ij^{*}}\left(h\right)$$\Xi_{r,i}\left(t+1\right)=\Xi_{r,i}\left(t\right)-\left(B_{h}t_{h}+B_{f}\frac{d_{f,ij^{*}}\left(t\right)\;}{v_{f}}+\frac{P_{tx}L_{D}}{\Psi_{ij^{*}}\left(t\right)}\right)$
**END For**

#### 4.3.2. Proposed BA-TS Algorithm 

The policy of TS depends on a pure Bayesian strategy, in which the rewards are assumed to be drawn from a predefined probabilistic model. TS is known to have excellent empirical performance even better than that achieved by UCB. A prior distribution is assumed for the rewards based on initializing the parameters of the said model. Then, during the learning process, the TS policy keeps track of the rewards’ posterior distribution using the collected data and then randomly pulls the arm, matching the probability of being optimal. Specifically, at each time t, random samples are taken from the constructed posterior distributions of the rewards and then select the arm with the maximum sample value to play. Then, the posterior distribution of the selected arm is updated via updating its model parameters for the next round of arm selection. Algorithm 2 describes the proposed BA-TS implemented in each access UAV *i*, where $\Psi_{ij}\left(t\right)$ is assumed to be drawn from Gaussian distribution, i.e., $N\left(E\left(\Psi_{ij}\left(t\right)\right),\sigma_{i,j}^{2}\left(t\right)\right)$, where $E\left(\Psi_{ij}\left(t\right)\right)$ and $\sigma_{i,j}^{2}\left(t\right)$ are the mean and variance of the distribution [25]. The assumption of Gaussian distribution is reasonable for the achievable data rate $\Psi_{ij}\left(t\right)$ because the received power has a normal distribution in nature due to the effect of additive white Gaussian noise (AWGN) and interferences. Following the methodology given in [26], $E\left(\Psi_{ij}\left(t\right)\right)$ and $\sigma_{i,j}^{2}\left(t\right)$ are set to $\frac{1}{x_{ij}\left(t\right)}\overset{x_{ij}\left(t\right)}{\underset{h=1}{\sum }}\Psi_{ij}\left(h\right)$ and $\frac{1}{x_{ij}\left(t\right)+1}$, respectively, where $x_{ij}\left(t\right)$ is the number of times gateway UAV *j* has been selected until time t. A prior Gaussian distribution is assumed at the beginning of the proposed BA-TS algorithm by initializing the values of $E\left(\Psi_{ij}\left(t\right)\right)$ and $\sigma_{i,j}^{2}\left(t\right)$ as given in Algorithm 2. Then at each time t, a sample $\phi_{ij}\left(t-1\right)$ is taken from each preconstructed posterior distribution of the data rate achieved from connecting access UAV *i* with gateway UAV *j*. 

Then, the gateway UAV $\;j^{*}$, which maximizes the following equation will be selected by access UAV *i*:

(9) $$j^{*}\left(t\right)=\underset{}{\arg\;\underset{1\leq j\leq M}{\max}\left(\phi_{ij}\left(t-1\right)-\rho \frac{d_{f,ij}\left(t\right)\;}{\Xi_{r,i}\left(t\right)\;}\right),\;}$$

where the battery cost term given in (8) is added to the maximization equation. Thus, the gateway UAV $j^{*}$, which has higher previous value of $\phi_{ij}\left(t-1\right)$ and requires lower battery cost of access UAV flight, will be selected by access UAV *i* at time *t*. After selecting gateway UAV $\;j^{*}$, access UAV *i* will fly and connect with it. Then, its achievable data rate $\Psi_{ij^{*}}\left(t\right)$ is observed and its corresponding number of selections is updated, as given in step 2 in Algorithm 2. Additionally, the parameters of the posterior Gaussian distribution corresponding to its reward distribution and remaining battery capacity are updated as well, as given in steps 3, 4, and 5 in Algorithm 2.
**Algorithm 2:** BA-TS gateway UAV selection. **Initialization:**t = 0, $E\left(\Psi_{ij}\left(t\right)\right)=0$, $x_{ij}\left(t\right)=0$, $\sigma_{i,j}^{2}\left(t\right)=1$ **For**
t=1:T Sample $\phi_{ij}\left(t-1\right)$, 1≤j≤M from normal distributions $N\left(E\left(\Psi_{ij}\left(t-1\right)\right),\sigma_{i,j}^{2}\left(t-1\right)\right)$
Draw a gateway UAV and obtain the reward:$j^{*}\left(t\right)=\underset{}{\arg\;\underset{1\leq j\leq M}{\max}\left(\phi_{ij}\left(t-1\right)-\rho \frac{d_{f,ij}\left(t\right)\;}{\Xi_{r,i}\left(t\right)\;}\right)}$Obtain $\Psi_{ij^{*}}\left(t\right)$
$x_{ij^{*}}\left(t\right)=x_{ij^{*}}\left(t-1\right)+1$$E\left(\Psi_{ij^{*}}\left(t\right)\right)=\frac{1}{x_{ij^{*}}\left(t\right)}\overset{x_{ij^{*}}\left(t\right)}{\underset{h=1}{\sum }}\Psi_{ij^{*}}\left(h\right)$$\sigma_{i,j^{*}}^{2}\left(t\right)=\frac{1}{x_{ij^{*}}\left(t\right)+1}$$\Xi_{r,i}\left(t+1\right)=\Xi_{r,i}\left(t\right)-\left(B_{h}t_{h}+B_{f}\frac{d_{f,ij^{*}}\left(t\right)\;}{v_{f}}+\frac{P_{tx}L_{D}}{\Psi_{ij^{*}}\left(t\right)}\right)$
**END For**

#### 4.3.3. Proposed BA-EXP3 Algorithm

EXP3 is a weighted MAB algorithm, where a weight is assigned to each arm, and the action is taken randomly with a probability proportional to the designated weights. Algorithm 3 gives the proposed battery-aware EXP3 gateway selection algorithm implemented in each access UAV *i*. In this algorithm, the weights are initialized to 1 for all available gateway UAVs then updated based on the weighted estimated rewards. The learning rate parameter of the algorithm, i.e., δ(t), and the exploration parameter χ∈(0,1] are also initialized. In the proposed BA-EXP3, the battery cost factor $\rho \frac{d_{f,ij}\left(t\right)\;}{\Xi_{r,i}\left(t\right)\;}$ is added to the weights of the of gateway UAVs as follows:

(10) $$w_{ij}\left(t\right)=w_{ij}\left(t\right)-\rho \frac{d_{f,ij}\left(t\right)\;}{\Xi_{r,i}\left(t\right)\;},$$

This means that the weights are updated based on both the estimated rewards and the battery cost function, as shown in Algorithm 3. Based on the weight factors, the probabilities of selecting gateway UAV *j* at time t, $\Pi_{ij}\left(t\right)$, are evaluated. Then, the gateway UAV $j^{*}$ is drawn randomly based on these probabilities as follows:

(11) $$j^{*}\left(t\right)\sim \Pi_{ij}\left(t\right)=\left(\Pi_{i1}\left(t\right),\;\Pi_{i2}\left(t\right),\ldots ,\;\Pi_{iM}\left(t\right)\right)$$

Access UAV *i* will fly and connect with gateway UAV $\;j^{*}$. After observing the actual payoff, i.e., $\Psi_{ij^{*}}\left(t\right)$, its weighted reward estimated value ${\hat{\Psi }}_{ij^{*}}\left(t\right)=\frac{\Psi_{ij^{*}}\left(t\right)}{\Pi_{ij^{*}}\left(t\right)}$ is calculated. In EXP3, dividing the actual gain by the probability that the action was selected compensates the payoff of actions that are unlikely to be selected. Then, the weights of both the selected gateway UAV and the other gateways are updated following the same methodology presented by the authors in [26], as given in steps 6 and 7 in Algorithm 3. Finally, the remaining battery capacity of the selected gateway UAV $j^{*}$ is updated as given in step 8. In the proposed algorithm, a time-dependent learning rate of $\delta \left(t\right)=\frac{\delta_{0}}{\sqrt{t}}$ is utilized, as given in [26]. The use of a time-dependent learning rate comes from the fact that large values of δ result in a more confident update, while small values of δ lead to conservative behavior [26].
**Algorithm 3:** BA-EXP3 gateway UAV selection.**Initialization:**$t=0,\;\delta \left(t\right)=\delta_{0},w_{ij}\left(t+1\right)=1$ for ∀j, χ **For**
t=1:T
$w_{ij}\left(t\right)=w_{ij}\left(t\right)-\rho \frac{d_{f,ij}\left(t\right)\;}{\Xi_{r,i}\left(t\right)\;}$$\Pi_{ij}\left(t\right)\leftarrow \left(1-\chi \right)\frac{w_{ij}\left(t\right)}{\sum_{j=1}^{M}w_{ij}\left(t\right)}+\frac{\chi }{M}$Draw a gateway UAV and obtain the reward:$\;j^{*}\left(t\right)\sim \Pi_{ij}\left(t\right)=\left(\Pi_{i1}\left(t\right),\;\Pi_{i2}\left(t\right),\ldots ,\;\Pi_{iM}\left(t\right)\right)$Obtain $\Psi_{ij^{*}}\left(t\right)$${\hat{\Psi }}_{ij^{*}}\left(t\right)=\frac{\Psi_{ij^{*}}\left(t\right)}{\Pi_{ij^{*}}\left(t\right)}$$\delta \left(t\right)=\frac{\delta_{0}}{\sqrt{t}}$$w_{ij^{*}}\left(t+1\right)={\left(w_{ij^{*}}\left(t\right)\right)}^{\frac{\delta \left(t\right)}{\delta \left(t-1\right)}}\exp\left(\delta \left(t\right)E\left(\Psi_{ij^{*}}\left(t\right)\right)\right)$$w_{ij}\left(t+1\right)={\left(w_{ij}\left(t\right)\right)}^{\frac{\delta \left(t\right)}{\delta \left(t-1\right)}},\forall j\neq j^{*}\;$$\Xi_{r,i}\left(t+1\right)=\Xi_{r,i}\left(t\right)-\left(B_{h}t_{h}+B_{f}\frac{d_{f,ij^{*}}\left(t\right)\;}{v_{f}}+\frac{P_{tx}L_{D}}{\Psi_{ij^{*}}\left(t\right)}\right)$
**END For**

## 5. Numerical Analysis

In this section, extensive numerical simulations are conducted to compare the performances of the proposed BA-UCB, BA-TS, and BA-EXP3 MAB algorithms for gateway UAV selection. Moreover, their performances are compared with two benchmark approaches based on near and random gateway selections. In the first approach, an access UAV always selects the nearest gateway UAV to it, while in the second one, a random gateway UAV is selected by the access UAV at every time. These two approaches are chosen as benchmarks because no prior information about the achievable data rates of the access–gateway–cellular links is required, making them practical solutions to the considered gateway selection problem. Other solutions based on exhaustive search, graph-based, branch-and-bound are impractical from the perspective of access UAV battery consumptions and gateway selection times. This is because, in these schemes, the achievable data rates of candidate access UAVs–gateway UAVs configurations should be known before choosing the optimal setting. However, these values are unknown unless access UAVs fly and connect with the gateway UAVs in a particular candidate configuration, making them unfeasible solutions. 

A post-disaster area of dimension 750 × 750 m^2^ is assumed where access UAVs are uniformly distributed inside this area for rescue services. Gateway UAVs are uniformly distributed around this area in a circle of 1250 m diameter. Based on (1), the minimum distance for establishing a mmWave communication link between an access UAV and a gateway UAV is equal to:

(12) $$d_{\min}={\left(\frac{P_{tx}G_{\max}^{2}{\left(\frac{\lambda }{4\pi }\right)}^{2}}{P_{rx}^{\mathrm{th}}}\right)}^{\frac{1}{\alpha }},$$

where $G_{\max}=\frac{2\pi -\left(2\pi -\theta_{-3\mathrm{dB}}\right)\varepsilon }{\theta_{-3\mathrm{dB}}}$ indicates the maximum antenna gain for a particular value of $\theta_{-3\mathrm{dB}}$. $P_{rx}^{\mathrm{th}}=-78\mathrm{dBm}$ is the threshold received power corresponding to modulation index 0 (MC0) of IEEE 802.11ad standard [44]. Thus, for example, when $\theta_{-3\mathrm{dB}}$ is equal to 10°, 20°, 30°, 40°, 50° and 60°, $d_{\min}$ becomes 357, 179, 120, 90, 72 and 60 m, respectively. At low values of $\theta_{-3\mathrm{dB}}$, long minimum distance,$\;d_{\min}$, can be held due to the free space propagation. Thus, the minimum flying distance by access UAV *i* towards gateway UAV *j* at time t is equal to:

(13) $$d_{f\min,ij}\left(t\right)=\left|d_{ij}\left(t\right)-d_{\min}\right|,$$

where $d_{ij}\left(t\right)$ indicates the radial separation distance between access UAV *i* and gateway UAV *j*. Table 1 summarizes the simulation parameters used throughout numerical simulation unless otherwise stated. It is assumed that all access UAVs are fully charged at the beginning of the game with a total battery capacity of $\Xi_{C}$ given in Table 1. 

### 5.1. Performance Metrics

We used the following metrics to assess the performances of the compared gateway selection schemes:

- **Average total system rate:** It is defined as the average sum rate of all UAVs relays over the time horizon. This can be expressed mathematically as:
  (14) $$R_{t}=\frac{1}{T}\underset{t}{\sum }\underset{i,j}{\sum }I_{ij}\left(t\right)\Psi_{ij}\left(t\right),$$
  where $I_{ij}\left(t\right)$ and $\Psi_{ij}\left(t\right)$ are defined in Section 5.
- **Average energy efficiency (bps/J) per access UAV:** It is defined as the average data rate of the access UAV divided by its total energy consumption. Total energy consumption of an access UAV *i* at time *t* is the sum of energy consumptions of the data communications, hovering, and flying. Thus, the average energy efficiency of an access UAV can be expressed as:
  (15) $$\Gamma =\frac{1}{T}\underset{t}{\sum }\frac{1}{N}\underset{i,j}{\sum }I_{ij}\left(t\right)\left(\frac{\Psi_{ij}\left(t\right)}{\Xi_{ij,h}\left(t\right)+\Xi_{ij,f}\left(t\right)+\Xi_{ij,c}\left(t\right)}\right),$$
  where $\Xi_{ij,h}\left(t\right)=B_{h}t_{h}$, $\Xi_{ij,f}\left(t\right)=B_{f}\frac{d_{f,ij}\left(t\right)}{v_{f}}$ and $\Xi_{ij,c}\left(t\right)=\frac{P_{tx}L_{D}}{\Psi_{ij}\left(t\right)}$ represent the hovering, flying, and data communication energies consumed by access UAV *i* when linked with gateway UAV *j* at a time t.
- **Convergence rate of the proposed MAB algorithms:** This measures the speed of convergence of the different proposed MAB algorithms despite the adversarial setting and the selfish behavior of the access UAVs. Towards this end, the system rate of the proposed MAB algorithms is evaluated against the time horizon.

### 5.2. Simulation Results

In the following section, the performances of the proposed BA-MAB-based gateway selection schemes are assessed under different system settings based on the performance metrics mentioned above. 

#### 5.2.1. Average Total System Rate

In this part of the simulation results, we give the average total system rate performances in Gbps against different values of gateway UAVs, access UAVs, and beam-widths.

**Figure 4:**
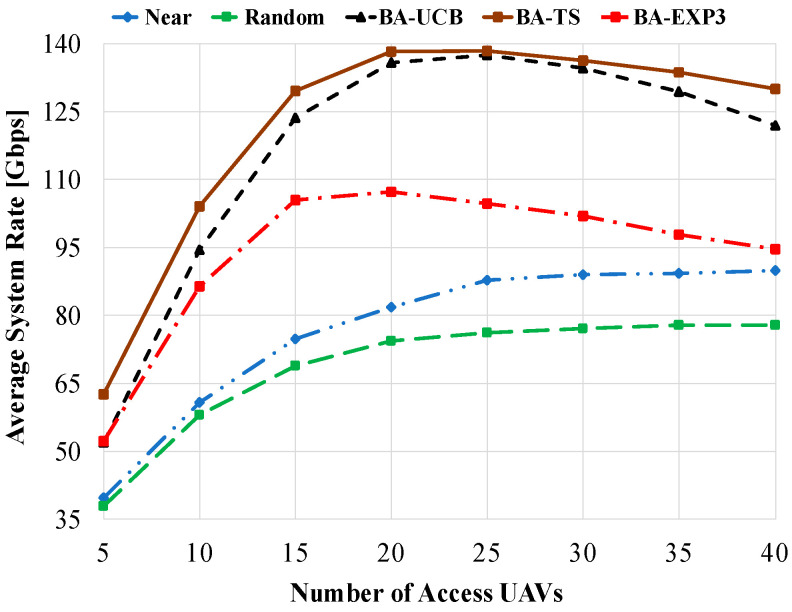
Average system rate against the number of access UAVs using gateway UAVs of 20 and a beam-width of 60°.

**Figure 5:**
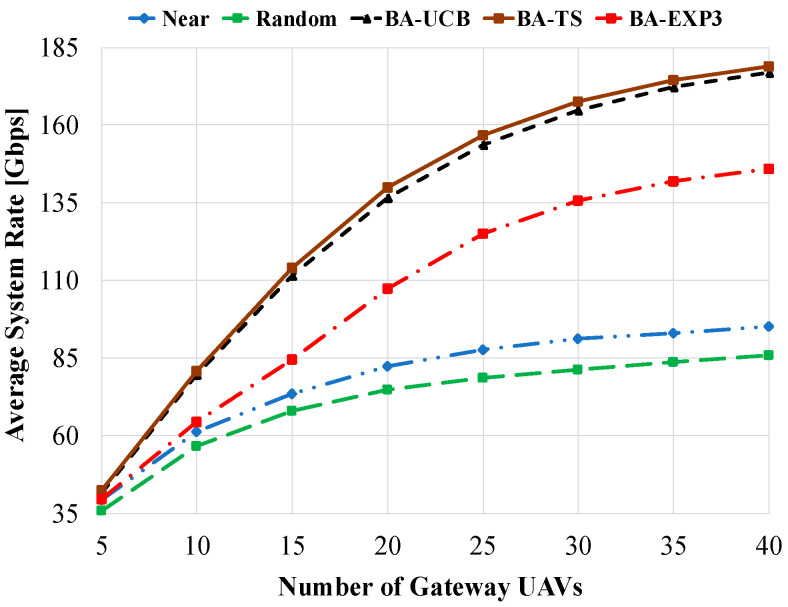
Average system rate against the number of gateway UAVs using access UAVs of 20 and a beam-width of 60°.

Figure 4 shows the average system rate of the compared schemes against the number of access UAVs using 20 gateway UAVs and a beam-width of 60°. As shown in this figure, the BA-TS has the best performance due to its integrated Bayesian strategy based on constructing posterior distributions for the obtained data rates. On the other side, random gateway selection has the worst performance due to the randomness in the selected gateway UAV at each round. Consequently, access UAVs will experience random interference as well as a random number of time slots at each time t. The near gateway selection has better performance than random selection due to the fixed pattern of interference and the number of assigned time slots experienced by access UAVs at each time t. It is interesting to note that the average system rate of the MAB algorithms is increasing when using few numbers of access UAVs until reaching a certain point, then slightly decreasing as the number of access UAVs is increased. This comes from the low interference and time-sharing scheduling experienced by the small number of access UAVs. However, as the number of access UAVs is increased beyond the number of gateway UAVs, i.e., 20 UAVs, high interference, and low number of time slots are experienced by access UAVs. Although all MAB algorithms are highly affected by interference at a higher number of distributed access UAVs, BA-TS and BA-UCB still have the best average system rate performances. From Figure 4, BA-EXP3 shows poor performance compared to the other MAB schemes and tends to reach the performance of the near selection at a high number of access UAVs. This comes from the nearly equal weights assigned to the gateway UAVs by the BA-EXP3 algorithm at each time step, which produces a poor gateway UAV selection policy. However, the BA-EXP3 algorithm still performs better than near and random selections. Using 25 access UAVs, BA-TS, BA-UCB, and BA-EXP3 have 60% (81%), 59.5% (80.5%), and 19% (37%) enhancement in the average system rate over the near (random) gateway selection, respectively. Figure 5 shows the average system rate against increasing the number of gateway UAVs using 20 access UAVs and a beam-width of 60°. For all compared schemes, as the number of gateway UAVs is increased, the average system rate is increased due to the decrease in the interference experienced by the access UAVs. Moreover, as a low number of access UAVs are linked with the same gateway UAV, more time slots are assigned to them, contributing to increasing the total system rate as well. Yet, BA-TS and BA-UCB have the best performances over the other schemes. It is also interesting to note that at interfering environments that are too harsh and at a low number of assigned time slots, e.g., when the number of gateway UAVs is equal to 5, MAB-based algorithms still have some improvements over near and random selections. However, as the number of gateway UAVs reaches 40, about 88% (108%), 86% (105%), and 54% (70%) increases in average system rates are obtained using BA-TS, BA-UCB, and BA-EXP3 overusing near (random) selection, respectively. 

**Figure 6:**
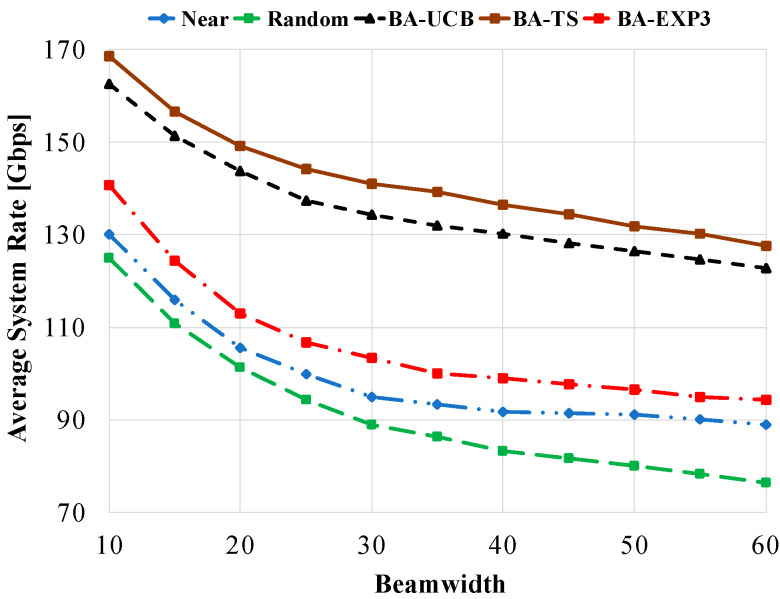
Average system rate against beam-width using access UAVs of 40 and gateway UAVs of 20.

Figure 6 shows the average system rate against the used beam-width using 20 gateway UAVs and 40 access UAVs. Generally, at lower values of beam-width, e.g., 10°, higher beam-forming gain and lower mutual interference occur, which highly increases the average system rate of all compared schemes. However, at higher values of beam-width, the beam-forming gain is decreased while the mutual interference is increased, resulting in a lower average system rate performance. From Figure 6, BA-TS has the best performance overall compared schemes, while random selection has the worst overall values of beam-widths due to the reasons mentioned above. At a beam-width of 10°, BA-TS, BA-UCB, and BA-EXP3 have 30% (34%), 25% (30%), and 8% (13%) increases in the average system rate over near (random) selection, respectively. However, at a beam-width of 60°, 43% (66%), 38% (61%), and 5% (23%) improvement is obtained. This emphasizes the superior performance of the proposed MAB algorithms, even in a high interfering environment.

#### 5.2.2. Average Energy Efficiency 

**Figure 7:**
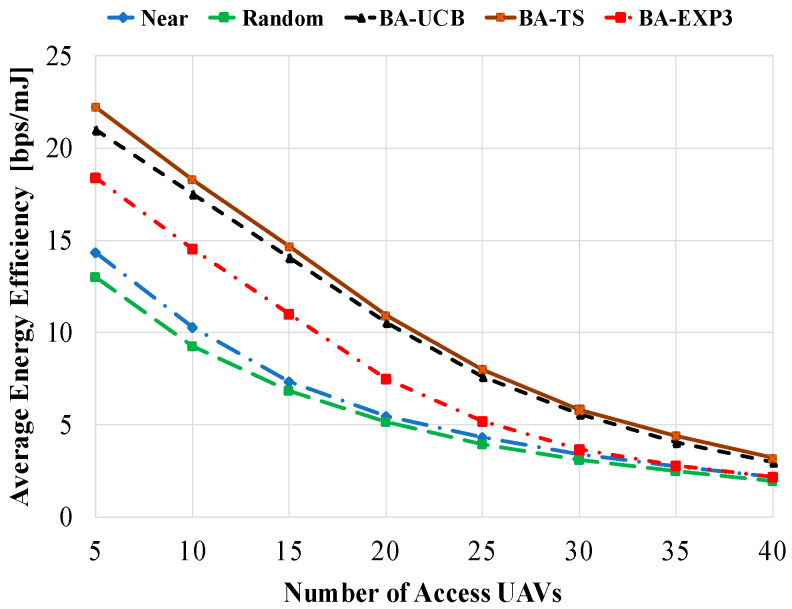
Average energy efficiency against the number of access UAVs using gateway UAVs of 20 and a beam-width of 60°.

In this part of simulation results, we study the average energy efficiency in bps/mJ of the compared gateway selection schemes against different values of gateway UAVs, access UAVs, and beam-widths. Figure 7 shows the average energy efficiency against the number of access UAVs using 40 gateway UAVs and a beam-width of 60°. As given in Figure 7, the proposed MAB-based gateway selection algorithms have better energy efficiency performances than near and random selections at all tested access UAV values. This comes from the proposed design of the BA-MAB algorithms, where the battery cost of the access UAV flight is taken into consideration while selecting the gateway UAV, maximizing its achievable data rate. For a low number of access UAVs, the data rate per access UAV is highly increased due to the low mutual interference and high number of assigned time slots. This results in high energy efficiency of all compared schemes, where the proposed BA-MAB algorithms show superior performances. However, at a high number of access UAVs, the achievable data rate of the access UAV is decreased due to the increase in the mutual interference accompanied by the decrease in the number of assigned time slots. This results in high decrease in the average energy efficiency, as shown in Figure 7. Yet, the BA-MAB algorithms show better performances than the other schemes. Using 5 access UAVs, about 60% (70%), 50% (62%), and 32% (42%) improvements in average energy efficiency are obtained using the proposed BA-TS-, BA-UCB-, and BA-EXP3-based gateway selections over near (random) selection, respectively. 

**Figure 8:**
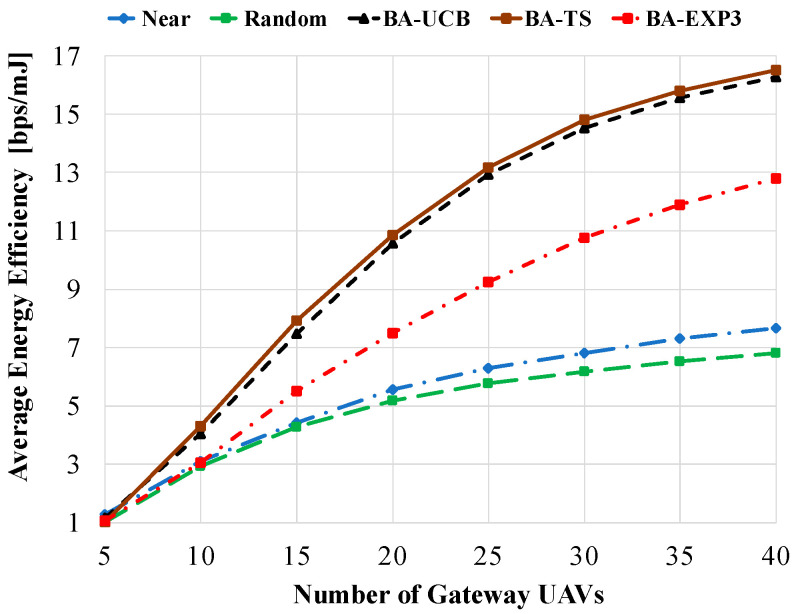
Average energy efficiency against the number of gateway UAVs using access UAVs of 20 and a beam-width of 60°.

Figure 8 shows the average energy efficiency against the number of gateway UAVs using 20 access UAVs and a beam-width of 60°. Due to the low achievable data rate per access UAV when using a small number of gateway UAVs, e.g., 5, the average energy efficiencies of all compared schemes are highly decreased, as shown in Figure 8. However, as the number of gateway UAVs is increased, the average energy efficiencies of all compared schemes are increasingly empowered by the increase in the achievable data rate per access UAV. At 40 gateway UAVs, 117% (143%), 114% (140%), and 68% (88%) enhancement in average energy efficiency is obtained using the proposed BA-TS, BA-UCB, and BA-EXP3 overusing near (random) selection, respectively.

**Figure 9:**
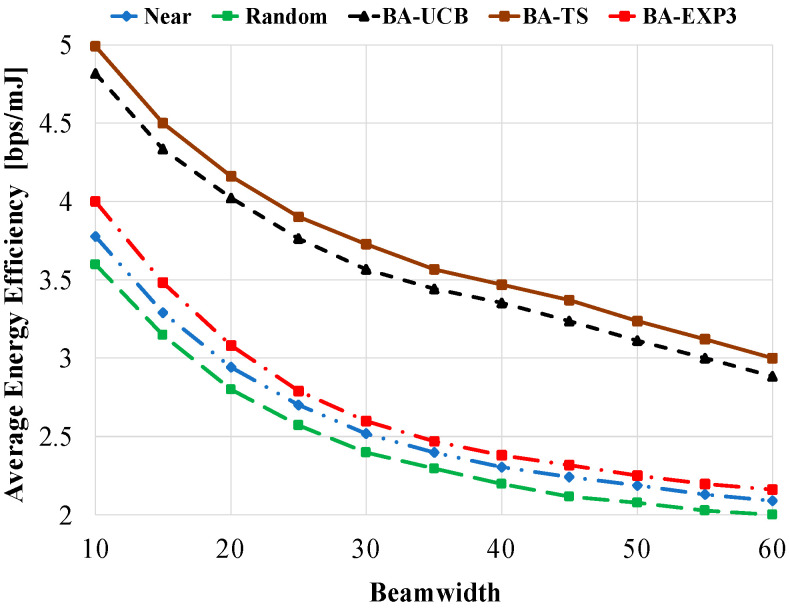
Average energy efficiency against beam-width using access UAVs of 40 and gateway UAVs of 20.

Figure 9 shows the average energy efficiency against the used beam-width using 20 gateway UAVs and 40 access UAVs. Influenced by the increase in the achievable data rate, the average energy efficiency of the access UAV is also increased at low values of beam-width, e.g., 10°. It is also decreased at high values of beam-width affected by the decrease in the achievable data rate, as previously explained. However, the proposed BA-MAB algorithms show better performances over the other compared schemes at all tested values of beam-width. At beam-width of 10°, about 33% (39%), 27% (33%), and 6% (11%) improvement in average energy efficiency is obtained using the proposed BA-TS, BA-UCB, and BA-EXP3 overusing near (random) selection, respectively. These values become 43% (50%), 37% (44%), and 2% (8%) at a beam-width of 60°. This confirms that the proposed BA-MAB algorithms show better performance even in high interfering environments.

#### 5.2.3. Convergence Rate

**Figure 10:**
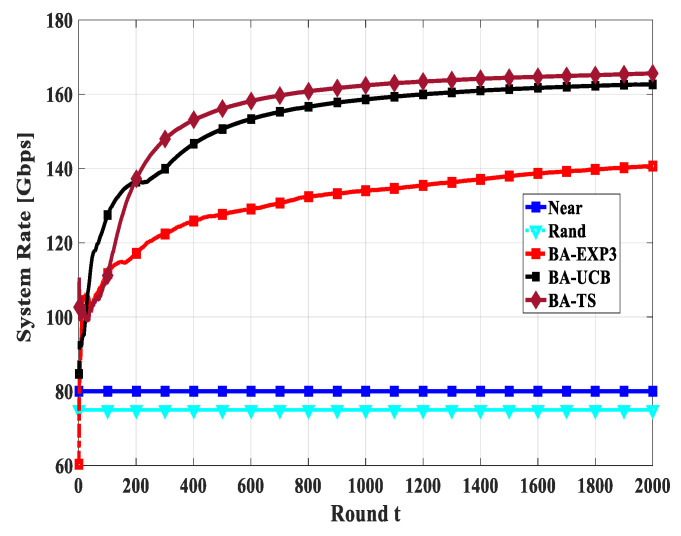
The convergence of system rate using access UAVs of 20, gateway UAVs of 20, and a beam-width of 60°.

**Figure 11:**
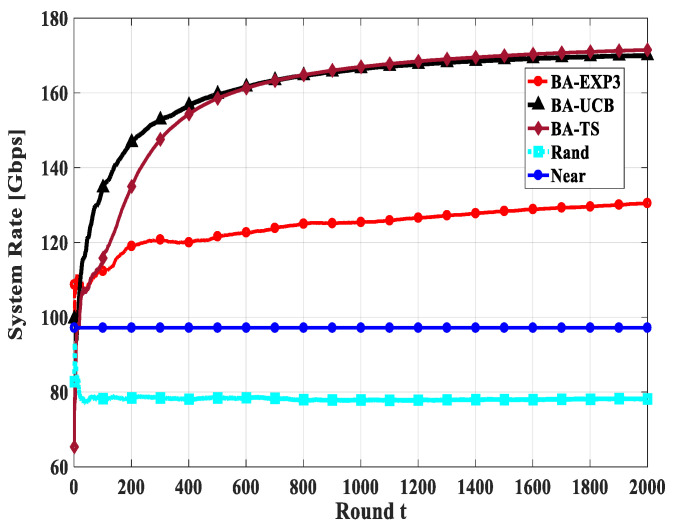
The convergence of system rate using access UAVs of 30, gateway UAVs of 20, and a beam-width of 60°.

**Figure 12:**
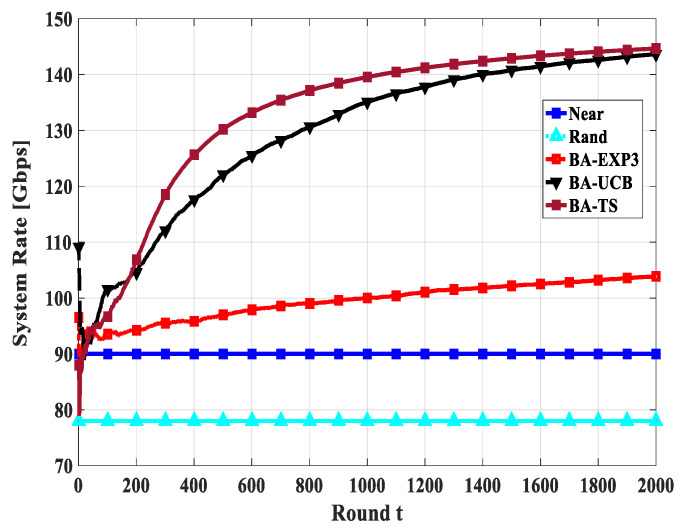
The convergence of system rate using access UAVs of 40, gateway UAVs of 20, and a beam-width of 60°.

Convergence is one of the primary metrics for MAB applications; the MAB algorithms should reach the sub-optimal solution using a few attempts. Thus, in this section, we study the convergence of the total system rate of the proposed BA-MAB algorithms in different settings. Figure 10, Figure 11 and Figure 12 show the convergence rate of the overall system rate using 20 gateway UAVs and a beam-width of 60° while changing the number of access UAVs by 20, 30, and 40, respectively. This emulates different interfering and time-sharing environments. In these figures, *t* indicates the rounds of gateway UAV selection not as an absolute value in seconds, as its absolute value will be different from round to round due to the different flight durations towards the selected gateway UAVs at each round of selection. From these figures, all proposed BA-MAB algorithms converged after a few trials; specifically, they start to converge after 400 rounds. These results demonstrate that the proposed BA-MAB algorithms can converge rapidly regardless of the adversarial setting of the problem and the selfish behaviors of the access UAVs. This means that access UAVs learn to play actions that enhance the overall system performance at every attempt.

## 6. Conclusions

In this paper, we considered the problem of gateway selection in a fully decentralized UAV wireless network. After formulating the optimization problem subject to its battery cost, we proposed a budget-constrained multi-player MAB algorithm to address the issue. Towards this end, three battery aware MAB (BA-MAB) algorithms were proposed, namely BA-TS, BA-UCB, and UA-EXP3. Due to the decentralized setting of the problem, selfish and concurrent multi-player BA-MAB algorithms were introduced, where each access UAV only relies on its previous observations when selecting the next gateway UAV. The proposed BA-MAB algorithms demonstrated superior performances over near and random gateway UAV selections in different environmental settings. Moreover, the MAB algorithms showed reasonable convergence rates. The obtained results open the door for applying ML techniques in general and MAB algorithms, especially for addressing several problems in UAV wireless networks. 

## Figures and Tables

**Table 1:** Simulation parameters.

Parameter	Value
$P_{tx}$	0.01 Watt (10 dBm) [13]
BW	2.16 GHz [13]
f	60 GHz [13]
α	2 [37]
$N_{0}$	−120 dBm
ε	0.01 [37]
$B_{h}$	4 Watt [36]
$B_{f}$	2 Watt [36]
$t_{h}$	120 s [36]
$v_{f}$	40 Km/h [36]
$\Xi_{C}$	400,000 Joule
ρ	1
$\delta_{0}$	0.1 [26]
χ	0.02
$L_{D}$	10 Gbps
